# Assessment of Technical and Clinical Success of Percutaneous Transhepatic Biliary Drainage for Postoperative Bile Leaks: A Favorable Alternative to Early Surgical Revision?

**DOI:** 10.7759/cureus.109240

**Published:** 2026-05-19

**Authors:** Mohammed Misbahuddin-Leis, Hamzah Adwan, Kristina Dubas, Thomas Mueller, Oleg Vorontsov, Christian Graeb, Boris Radeleff

**Affiliations:** 1 Radiology, Sana Klinikum Hof GmbH, Hof, DEU; 2 Medical Faculty Heidelberg, Heidelberg University, Heidelberg, DEU; 3 Clinic for Radiology and Nuclear Medicine, Goethe University Frankfurt, Frankfurt, DEU; 4 Department of Diagnostic and Interventional Radiology, Sana Klinikum Hof GmbH, Hof, DEU; 5 Department of Gastroenterology, Hepatology, Infectiology, Hematology and Oncology, Sana Klinikum Hof GmbH, Hof, DEU; 6 Department of Visceral and Abdominal Surgery, Sana Klinikum Hof GmbH, Hof, DEU; 7 Department of Surgery, Sana Klinikum Hof GmbH, Hof, DEU

**Keywords:** bda, biliary leakage, cystic stump, escalation strategy, insufficiency, ptbd

## Abstract

Purpose

This study aimed to evaluate the role of percutaneous transhepatic biliary drainage (PTBD) with an escalation strategy for managing postoperative bile leaks as an alternative to early surgical revisions.

Methods

We retrospectively reviewed 24 patients (14 males, 10 females; mean age 69.3 ± 11.7 years, range 45-93) transferred by the Department of Visceral and Abdominal Surgery between January 2018 and October 2021. Twenty-three PTBDs were placed, followed by 107 follow-up biliary interventions using an escalation strategy that involved progressively larger drains (8, 10, 12F) and modified PTBDs with side holes avoiding the insufficiency area.

The primary endpoints were technical and clinical success. Technical success was defined as successful PTBD placement with confirmed bile drainage. Clinical success was determined by bile leak resolution, assessed through bile output reduction, normalization of inflammatory markers, and imaging confirmation.

Results

A total of 141 interventional procedures were performed, including 23 initial PTBD insertions, 107 follow-up controls, and 10 additional ultrasound- or CT-guided drainages. For successfully treated patients, the interventional therapy averaged 72.5 ± 76.2 days, ranging from nine to 239 days. Technical success was 100% in both groups. Clinical success was 93.8% in the primary PTBD group and 14.3% in the secondary PTBD group.

Conclusion

Our retrospective descriptive case series describes the use of PTBD for postoperative bile leaks, with high technical success and favorable clinical outcomes observed - particularly among patients who received PTBD as the primary intervention. In contrast, patients who underwent PTBD after failed surgical revision appeared to experience higher mortality and more postoperative complications.

## Introduction

The creation of a bilio-digestive anastomosis (BDA) is one of the most challenging aspects of major abdominal surgeries, such as radical pancreatoduodenectomy or liver resections, due to the risk of postoperative BDA insufficiency (0-5%) [1] or cystic duct stump closure insufficiency (0.3-3%) [2]. BDA insufficiency is a serious complication after hepatopancreatic surgery, potentially leading to biliomas, abscesses, peritonitis, sepsis, liver failure, vascular complications, and death [3-6]. Common causes include inadequate surgical durability (e.g., insufficient suture stability or excessive anastomotic tension), unexpected injuries (e.g., accidental bile duct damage during dissection), or compromised blood supply (e.g., ischemia due to vascular injury or devascularization) [3].

Diagnosis of a bile leak is indicated by biliary discharge through surgical drains, with typical signs being fever, abdominal pain, shoulder pain, and pleural effusion. While small bile leaks may resolve spontaneously, drainage exceeding 300 ml/24 hours in the first three postoperative days typically requires intervention [7-9]. Surgical revision (relaparotomy) is indicated within three days for grade C bile leaks, but this is associated with high morbidity (22-37%) and mortality (3-18%) [10-13].

## Materials and methods

This retrospective descriptive case series evaluated the use of a structured escalation strategy involving percutaneous transhepatic biliary drainage (PTBD) for the management of postoperative bile leaks. Patients were grouped based on their treatment sequence: one group received PTBD as the primary intervention, while the other underwent initial surgical revision with Neuhaus drainage, followed by PTBD due to persistent biliary insufficiency. The escalation approach involved sequential upsizing of PTBD catheters (ranging from 8F to 12F), and in select cases, the addition of CT- or ultrasound-guided perihepatic drainages for perihepatic biliomas when indicated.

The primary outcomes assessed were technical and clinical success, defined by successful bile drainage and resolution of the leak, respectively. Secondary measures included the duration of intervention, the occurrence of complications, and patient survival during follow-up.

The outcomes of each group are presented separately to reflect observed differences in clinical course. While PTBD is a well-established intervention, detailed reports describing structured escalation strategies for postoperative bile leaks - particularly following failed surgical revision - remain limited. This case series adds to the descriptive literature and may help inform further management approaches in similar clinical scenarios.

**Figure 1:**
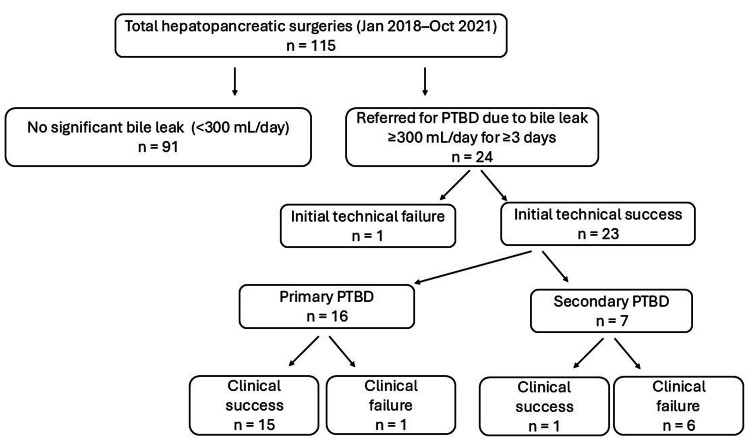
Flow diagram of patient inclusion. Data are presented as n (%) or mean ± standard deviation, unless otherwise specified. The total number of biliary surgeries between January 2018 and October 2021 was 115. Patients with minor bile leaks were managed conservatively. A total of 24 patients with persistent bile leak >300 ml/day for three or more days were referred for percutaneous transhepatic biliary drainage (PTBD) and included in the study.

We conducted a retrospective review of medical and radiological records for patients with postoperative bile leaks from the BDA (leakage from the anastomosis between the bile duct and small bowel), duodenal stump (leakage due to insufficiency after surgical duodenal closure), cystic stump (leakage due to insufficient cystic duct stump closure), and bile ducts (leakage due to bile duct injuries) who were internally referred by the Department of Visceral and Abdominal Surgery for PTBD treatment between January 2018 and October 2021. The median time from surgery to initial PTBD placement was 12.1 ± 7.3 days with a range of one to 28 days. Patients were referred to our interventional radiology department by the surgical team if they presented with clinically significant bile output of ≥300 ml/day for at least three consecutive days, prompting the need for percutaneous drainage. Bile leaks with lower output (<300 ml/day) were not referred, as they typically resolved spontaneously without requiring invasive intervention, as illustrated in Figure 1.

We recorded clinical data including patient outcomes, complications, comorbidities, American Society of Anesthesiologists (ASA) scores, site of bile leak, type and timing of the initial surgery, time interval between surgery and PTBD, PTBD duration, prior surgical or interventional procedures, procedural details, and magnetic resonance cholangiopancreatography (MRCP)/endoscopic retrograde cholangiopancreatography (ERCP) reports. While we acknowledge the presence of confounding variables in this retrospective setting, this information was used to contextualize patient outcomes during analysis. Data were sourced from the hospital information system (KIS, ORBIS™; Dedalus, Bonn, Germany) and imaging archives (PACS, Centricity™; GE Healthcare, Buc, France). Given the retrospective nature of this study, the sample size was determined by all eligible cases within the defined time frame.

The primary study endpoints were technical success, defined as successful placement of the PTBD catheter with adequate bile drainage confirmed by fluoroscopic cholangiography and ultrasound immediately after the procedure, and clinical success defined complete resolution of bile leakage, determined by firstly reduction of bile output to <50 ml/day over at least two consecutive 24-hour periods, secondly normalization of laboratory parameters, particularly bilirubin levels and C-reactive protein (CRP), thirdly absence of clinical symptoms related to bile leakage, such as fever, pain, or peritonitis and finally complete closure of the bile leak, confirmed by PTBD control imaging. Patients were considered to have failed therapy if bile leakage persisted despite PTBD and additional interventions (e.g., surgical revision or re-drainage), or if they died due to bile leak-associated complications.

**Figure 2:**
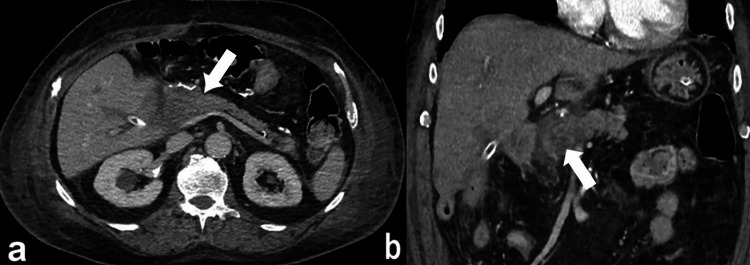
Typical CT findings a, b: Contrast-enhanced multidetector computed tomography (MDCT) scan performed on postoperative day three in a patient following pylorus-preserving pancreaticoduodenectomy due to bile output >300 ml/day from the surgical drain and signs of systemic inflammation. A subtle rim of fluid collection is seen around the bilio-digestive anastomosis (BDA), raising suspicion of an anastomotic insufficiency (white arrow).

Bile leakage (>300 ml daily) within three postoperative days was suspected based on clinical symptoms, liver function tests, and imaging (CT) [14-18]. Typical CT findings included fluid collections around the BDA, with or without fat stranding, free air near the leak, and biliary duct changes, as illustrated in Figure 2A-2B.

Initial PTBD procedures were performed under sedation using midazolam and pethidine hydrochloride. For uncooperative or ICU patients, general anesthesia was administered. A standardized ultrasound/fluoroscopy-guided PTBD technique [18,19] involved puncturing the liver lobe with a 22G needle (Neff Percutaneous Access Set™; Cook Medical, Bloomington, IN, USA) under ultrasound guidance, followed by cholangiography and the "double needle technique" to access the biliary branch. In all cases, PTBD was performed via a right-sided approach by experienced, board-certified interventional radiologists (five or more years of experience). The approach was selected based on anatomical accessibility and institutional protocol.

**Figure 3:**
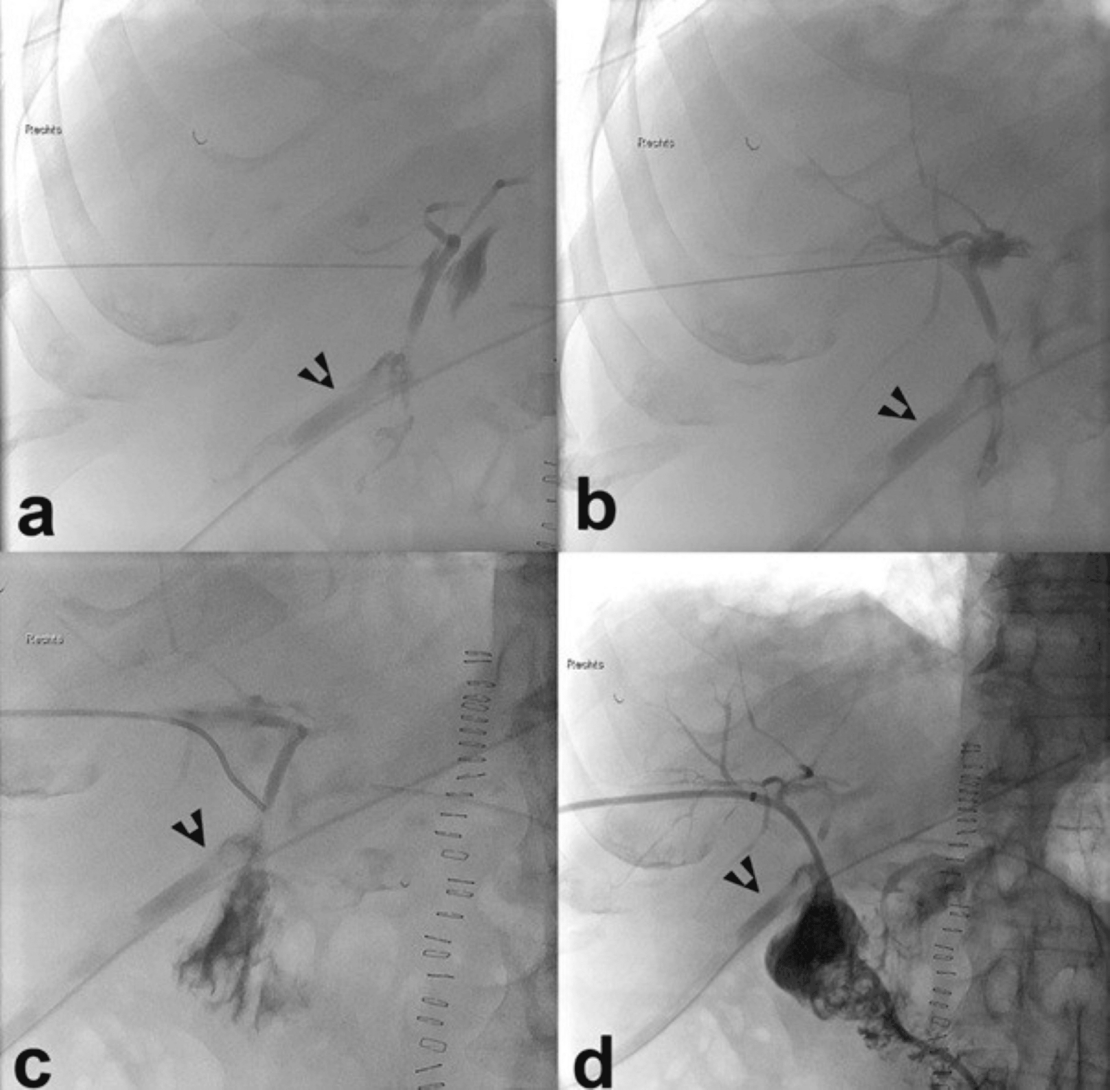
Percutaneous transhepatic biliary drainage (PTBD) placement following clinical suspicion of biliodigestive anastomotic insufficiency (bile output >300 ml/day via surgical drainage). a–b: After sonographically and fluoroscopically guided puncture of the right biliary system using a Neff set (Cook Medical, Bloomington, IN, USA), contrast injection reveals a clear leak from the common bile duct (CBD) with immediate extravasation into the surgical drainage (black arrowhead). c–d: Successful probing of the biliary tree using a 4F Berenstein catheter (Cordis, Miami Lakes, FL, USA) and a 0.035″ hydrophilic guidewire (Terumo, Tokyo, Japan), followed by insertion of an 8F pigtail-configured drainage catheter (Cook) over a stiff 0.035″ wire (Amplatzer Super Stiff, Boston Scientific, Marlborough, MA, USA). Final placement is confirmed, and the drain is secured subcutaneously.

**Figure 4:**
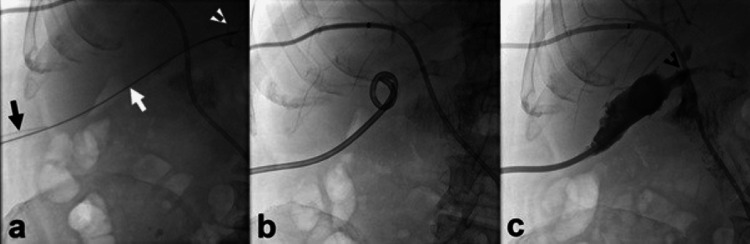
Interventional management following accidental removal of a surgically placed Robinson drain. a: Five days postoperatively, the patient accidentally dislodged the Robinson drain. The remaining 1 cm tract was probed using a guidewire (black arrow; Terumo, Tokyo, Japan), followed by catheter advancement. A 0.035″ super stiff wire (white arrow; Amplatzer Super Stiff, Boston Scientific, Marlborough, MA, USA) was then inserted. b: After wire access, the Robinson drain was removed. A 12F pigtail drain (Boston Scientific, USA) was advanced into the periductal bilioma cavity adjacent to the site of insufficiency. c: Contrast injection through the percutaneous transhepatic biliary drainage (PTBD) revealed ongoing - though reduced - leakage from the common bile duct (CBD) into the biloma cavity now drained by the 12F catheter (black arrowhead).

**Figure 5:**
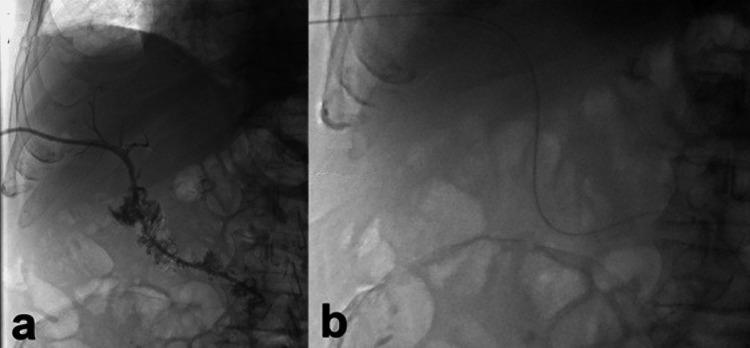
Follow-up percutaneous transhepatic biliary drainage (PTBD) evaluation and drainage step-down. a: Three weeks after the previous intervention, contrast imaging was performed through an 8F Yamagawa drainage catheter (Cook Medical, Bloomington, IN, USA). The imaging showed no residual biliary leakage, confirming resolution of the anastomotic insufficiency. b: The drainage was subsequently downsized to a shortened 5F pigtail catheter, used as a placeholder to facilitate potential upsizing to an 8F Yamagawa catheter should bilirubin levels rise again.

**Figure 6:**
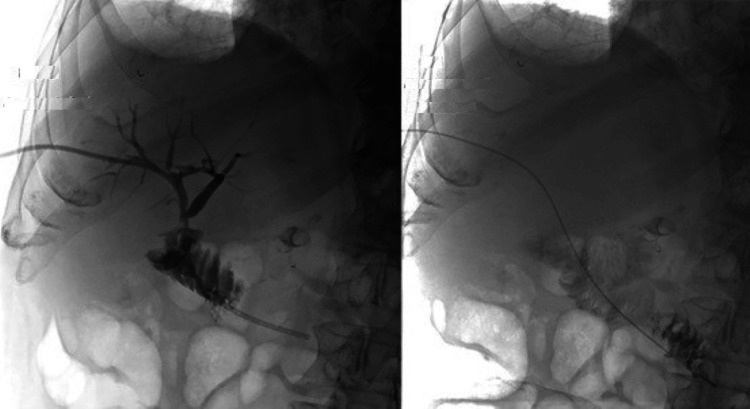
Final step-down and successful conclusion of percutaneous transhepatic biliary drainage (PTBD) therapy. Left: After 1.5 months of sustained drainage, the 8F Yamagawa (Munich) catheter (Cook Medical, Bloomington, IN, USA) was successfully removed. A 5F pigtail catheter was inserted as a placeholder to allow for further short-term monitoring. Right: Final fluoroscopic control after three days showed no signs of recurrent leakage. The placeholder catheter was removed without complication, marking the end of therapy.

A 0.018-inch guidewire facilitated the placement of a 5F sheath, followed by biliary system recanalization using a 0.038-inch guidewire (Guide Wire M, Radiofocus™; Terumo, Tokyo, Japan) and a 4Fr/65cm Berenstein catheter. The Neff sheath was removed, and the puncture site was dilated for PTBD insertion. An 8.5F biliary drainage catheter (Ultrathane®; Cook, or BiliarPlus®Hydro F; Peter Pflugbeil GmbH, Zorneding, Germany) was positioned, and cholangiography confirmed placement as illustrated in Figures 3A-3D, 4A-4C, 5A-5B, 6.

Finally, the PTBD is fixed subcutaneously using a double-knot technique to avoid later dislocation of the drainage due to mishandling on the surgical ward [20].

Antibiotic prophylaxis (intravenously administered Unacid 3g) was administered, with bile samples collected for antibiogram analysis. Routine saline irrigation (10-15 ml) was recommended. Follow-up occurred within a week.

Bile leakage was monitored daily, and if unresolved after three to five days, the escalation strategy (8F, 10F, and 12F drains) was implemented. As a maximum, a modified 12F abscess drain with manually made peripheral side holes was utilized, sparing the insufficiency area. In cases of perihepatic biliomas identified on initial or follow-up imaging, an additional percutaneous (ultrasound- or CT-guided) drainage was placed for optimal biliary decompression.

## Results

Statistics

Patient characteristics, including age, gender, treatment group (primary vs. secondary PTBD), clinical outcomes, and complications, were collected and summarized descriptively. Categorical variables are presented as counts (n) and percentages (%), while continuous variables are reported as means with standard deviations or as ranges where appropriate. Bile concentrations and CRP serum levels were also summarized using mean values. Given the small sample size of this study, no formal statistical comparisons were performed. All data handling and summary statistics were carried out using R software (version 4.1.1; R Foundation for Statistical Computing, Vienna, Austria) and RStudio (version 2021.9.0.351; Posit PBC, Boston, MA, USA).

Patient population and technical success

In total, for 24 patients (14 men, 10 women, mean age 71.9 ± 11.7 years), who were referred to our department for treatment of postoperative biliary insufficiency (see Table 1 for patient population analysis), we performed a total of 141 interventional procedures, including 23 initial PTBD insertions, 107 PTBD controls, and 10 additional ultrasound- or CT-guided drainage insertions of bilomas (one ultrasound-guided and nine CT-guided, Table 2).

**Table 1:** Patient population analysis Data are presented as n (%) or mean ± standard deviation, unless otherwise specified.

Indication for surgery	Surgical procedure	n (%)	Site of bile leak	n (%)
Pancreatic head tumor	Post-pylorus-preserving Whipple (pp-Whipple)	4 (16.7%)	Biliodigestive anastomosis	2 (8.3%)
Adenoma of the papilla of Vater	Post-pylorus-preserving Whipple (pp-Whipple)		"	1 (4.2%)
Bleeding duodenal ulcer	Post-pylorus-preserving Whipple (pp-Whipple)		"	1 (4.2%)
Bleeding duodenal ulcer	Classic pancreatoduodenectomy (Standard Whipple)	2 (8.3%)	"	2 (8.3%)
Adenocarcinoma of the esophageal junction	Total gastrectomy	2 (8.3%)	"	2 (8.3%)
Ileus due to covered perforated sigmoid carcinoma and carcinoma of the right colonic flexure	Open surgical colectomy	1 (4.2%)	"	1 (4.2%)
Adenocarcinoma of the pancreatic body	Total pancreatectomy	1 (4.2%)	"	1 (4.2%)
Intraductal papillary mucinous neoplasm	Classic pancreatoduodenectomy (Standard Whipple)	1 (4.2%)	"	1 (4.2%)
Perforated duodenal ulcer	2/3 gastric resection with Roux-en-Y reconstruction	1 (4.2%)	"	1 (4.2%)
Bleeding duodenal ulcer	2/3 gastric resection with Roux-en-Y reconstruction	3 (12.5%)	Duodenal stump	2 (8.3%)
Gastric ulcer perforation	2/3 gastric resection with Roux-en-Y reconstruction		"	1 (4.2%)
Gastric adenocarcinoma	Total gastrectomy	1 (4.2%)	"	1 (4.2%)
Cholecystitis	Cholecystectomy	5 (20.8%)	Cystic stump	4 (16.7%)
Gastric ulcer with adenocarcinoma	Cholecystectomy		"	1 (4.2%)
Hepatic metastasized rectal carcinoma	Segment 8 resection – segment 7 atypical resection	1 (4.2%)	Distal common bile duct	1 (4.2%)
Hepatic metastasized colorectal carcinoma	Segment 4–8 resection	1 (4.2%)	Bile duct	1 (4.2%)
Recurrent intrahepatic cholangitis with multiple liver abscesses	Bisegmentectomy (segments 2/3)	1 (4.2%)	"	1 (4.2%)
Total		24 (100%)		24 (100%)

**Table 2:** Additional ultrasound - & CT-guided drainage and size of drainage catheters Data are presented as n (%) or mean ± standard deviation, unless otherwise specified.

Size of drainage catheters	No. of patients (n, %)
6F	1 (10.0%)
8F	7 (70.0%)
10F	1 (10.0%)
12F	1 (10.0%)
Total	10 (100%)

Furthermore, in our study, patients were categorized into two groups based on their therapeutic interventions. In the primary PTBD group (n=16, 69.6%), PTBD was administered as the initial therapy of the biliary insufficiency without a surgical revision. The secondary PTBD group (n=7, 30.4%) underwent initial surgical revision for biliary insufficiency as the initial therapy, with the insertion of Neuhaus drainage (Sizes 8F-10F; PFM Medical AG, Cologne, Germany), followed later by PTBD as a rescue treatment due to failed surgical revision and ongoing biliary insufficiency.

Overall, the initial PTBD placement was technically successful in 23 (95.8%) patients (16.4±19.8 days after the operation, with a range of 3-77 days). PTBD placement was performed in general anesthesia by the anesthetist in eight (34.8%) patients. In the remaining 16 (69.6%) cases, analgosedation (under strict monitoring and oxygen mask, and i.v. hydration) was provided by radiologists as explained prior in the paper. In one patient, initial PTBD placement was technically unsuccessful due to a very steep puncture angle in a decompressed, tiny bile duct system. This patient presented again after three months with a significant increase of the preexisting as well as new multiple cholangitic abscesses, impaired liver perfusion due to thrombosis of the anterior pedicle of the right portal vein, and stenosis of the hepatic artery. We then performed a technically successful PTBD procedure on this patient. The patient died in the further course after 28 days after his re-presentation as a result of liver failure.

In summary, within the 107 PTBD controls, subsequent procedures comprised sole cholangiography, exchange with a new drainage or up- and downsizing. The most common were follow-ups for sole cholangiography (n=52) devoid of any intervention. We performed a routine exchange of a new 8F biliary drainage in 16 cases (mean 44±48.2 days with a range of 4-158 days), upsizing from 8 to 10F occurred in 16 cases (mean 16±25.7 days, with a range of 3-74 days), and upsizing from 10 to 12F biliary drainage was conducted in six cases (mean 31±45 days with a range of 3-83 days). Moreover, at the end of the therapy there were 17 interventions for downsizing the drainage to a 5F placeholder.

In the initial 24 PTBD procedures, there were a total of two major complications (two liver hematomas without the need for further interventions or consequences) observed, and no intervention-related mortality was recorded. In the subsequent 107 PTBD follow-up assessments, no major complications were noted. Minor complications, specifically postoperative cholangitis, were encountered in two cases (8.7%). For the 10 ultrasound- or CT-guided bilioma drainages (n=8, 34.8%), no complications occurred.

Clinical success

The postoperative biliary insufficiency was completely resolved in 16 (69.6%), and all these patients survived the post-interventional course with a follow-up of 79±32 days. In contrast, seven (30.4%) patients with a clinically persistent insufficiency died during the post-interventional course (haemorrhagic shock, n=1 (4.3%), as a result of tumor disease, n=1 (4.3%), due to septic shock, n=2 (8.7%), in multi-organ failure, n=3 (13%)). In one (4.3%) patient with ongoing insufficiency, a relaparatomy was performed after PTBD placement, with massive further increase in inflammatory parameters and increasing bilioma with placement of a T-drainage. Unfortunately, the patient passed away due to fulminant sepsis with acute respiratory distress syndrome (ARDS) and multiorgan failure 15 days after PTBD placement and 13 days after the surgical reintervention.

In all (n=16, 69.6%) clinically successfully treated patients, the entire interventional therapy (spanning from the initial PTBD to the conclusion of subsequent follow-up interventions) had an average duration of 72.5 ± 76.2 days, with a range of nine to 239 days. This timeframe was measured from the diagnosis of postoperative biliary insufficiency to the verification of its closure in the PTBD control.

Primary study endpoint: observed trends in clinical outcomes

Among patients in the primary PTBD group (n=16), a higher rate of clinical success (n=15, 93.8%) and lower mortality (n=1, 6.3%) were observed compared to those in the secondary PTBD group (n=7), who had undergone surgical revision prior to PTBD. Postoperative complications also appeared more frequent in the secondary group (clinical success n=1, 14.3%; mortality n=6, 85.7%). The deceased patient in the primary PTBD group initially presented with chronic cholecystitis and choledocholithiasis with cholecystoduodenal and cholecystocolic fistulas and underwent cholecystectomy complicated by cystic stump insufficiency, cholangitis, and fulminant sepsis with ARDS and multiorgan failure. The single survivor in the secondary PTBD group had duodenal stump insufficiency following gastric resection for perforated chronic duodenal ulcer disease and underwent multiple relaparotomies prior to PTBD placement. These descriptive observations suggest that patients receiving PTBD as the initial intervention experienced a more favorable clinical course than those requiring PTBD after failed surgical revision. 

Secondary study endpoints

Additional percutaneous CT- or ultrasound-guided drainages were used in both groups as part of the management strategy for perihepatic fluid collections. These procedures were required in seven (43.8%) patients in the primary PTBD group and in one (14.3%) patient in the secondary PTBD group, indicating that such additional interventions were more frequently required in the primary PTBD group.

Regarding laboratory markers, patients in the primary PTBD group showed a notable reduction in inflammatory and biliary parameters. Mean CRP levels decreased from 14.96 to 9.66, and bile levels dropped from 6.65 to 0.7. In contrast, in the secondary PTBD group, CRP levels also declined (from 18.63 to 10), but bile levels increased from 1.46 to 3.62 (Table 3), reflecting the more severe clinical condition and ongoing septic course in these patients.

**Table 3:** Laboratory data Comparison of mean pre- and post-interventional laboratory parameters (C-reactive protein (CRP) and bile levels) between the primary and secondary percutaneous transhepatic biliary drainage (PTBD) groups. Data are presented as mean values.

	Primary PTBD group		Secondary PTBD group	
	CRP before	CRP after	CRP before	CRP after
Mean	14.96	9.66	18.63	10
	Bile before	Bile after	Bile before	Bile after
Mean	6.65	0.7	1.46	3.62

## Discussion

The frequency and clinical impact of biliary leaks following hepatobiliary surgery depends upon the specific operation and clinical conditions of the patients. For example, the incidence of biliary leaks subsequent to laparoscopic cholecystectomy is consistently reported to be up to 2% [21,22]. In comparison, the documented incidence of bile leaks following cadaver liver transplantation stands at 4.3%. After major hepatic resection biliary leaks yield a range of 3% to an enormous 11% [23].

The clinical impact of biliary leaks can be severe, with documented 90-day mortality rates for bile leaks according to the International Study Group for Liver Surgery manifesting as 1.9% for grade A, 2.5% for grade B, and, notably, 25% for grade C [24].

The available treatment options for postoperative bile leaks include conservative measures, endoscopic biliary drainage (EBD), surgical revision, and percutaneous biliary drainage. The endoscopic approach with EBD, in most cases the first used, may face challenges in the context of BDA insufficiency, leading to procedural failure in 3% to 5% [25,26]. Anastomotic strictures, complete anastomotic disruption, complex anatomical changes, poor visualization due to scarring or inflammation, the presence of ascites, and technical difficulties in navigating altered anatomy contribute to unsuccessful EBD [27-30]. Additionally, the inability to pass or advance devices through severely narrowed or obstructed anastomotic sites, underlying diseases such as malignancies, and patient-related factors further impact the success of EBD. Suboptimal stent placement and the potential for stent-related complications can also contribute to the ineffectiveness of EBD in cases of BDA insufficiency [31,32]. Although ERCP with sphincterotomy and/or stenting is considered a standard treatment option for cystic stump leakage, PTBD was preferred in selected patients in our cohort due to persistent high-output leakage, cholangitis, or complex postoperative clinical conditions after interdisciplinary evaluation. However, the success of EUS-BD is also dependent on the endoscopist's skill and patient factors, and further research is needed to establish its efficacy as a standard technique.

Due to the development of EBD, surgery is no longer the first or only approach for bile leaks and biliodigestive complications due to the associated elevated morbidity and mortality rates [11,33-35]. Surgery is deemed necessary as definitive therapy when there is no bilio-enteric continuity, non-surgical methods with bilio-enteric continuity have proven ineffective, or when surgery is the primary treatment for an associated pathology, such as malignancy. Historically, as stated above, surgical revision of bile leaks has been associated with a high morbidity rate (22% - 37%) and mortality rate (3% - 18%).

As the third option, PTBD as a minimal-invasive therapeutic modality for managing biliary insufficiency post-surgery has progressed significantly over recent decades.

In 2010, Stampfl et al. [7] investigated the potential role of PTBD escalation therapy, as used in this study, as an early and definitive intervention instead of surgical revisional operations. At that time, they successfully treated bile duct system insufficiency in 22 (73.3%) patients with an average of 55.2 days (compared to n=16 (69.6%) patients in 72.5 days in our study). The survival rate in their study was 73.3%, while in our study, it was 69.6%. The major complication rate was low in both cohorts (6.6% in their study and 8.2% in ours).

We achieved in this study a complete resolution of postoperative bile duct insufficiency in all surviving patients (n=16, 69.6%) through escalating PTBD treatment, with an average duration of 72.5 days. All patients (n=7, 30.4%) without clinical success, indicating persistent insufficiency, died during the postinterventional course.

The central observation of this study is that clinical outcomes, including survival and complication rates, appeared to differ depending on whether patients received primary PTBD or secondary PTBD after confirmed biliary insufficiency. While Stampfl et al. previously explored this therapeutic question without statistical validation, our findings descriptively support the notion that PTBD, when performed in experienced hands, may be associated with a more favorable clinical course in selected patients.

Koch et al. reported that early postoperative bile leakage without prior radiologic imaging or intervention might require immediate surgical intervention and reconstruction of the BDA [8]. Grade C bile leakage is a severe complication that typically necessitates relaparotomy. This procedure involves suture closure of leaking bile ducts, clearance of intra-abdominal fluid collections, (re)construction of the bilioenteric anastomosis, and placement of additional drains for postoperative lavage. In contrast to these findings, we propose that even grade C bile leaks detected within the first three days, based on the volume of drain fluid and/or the bilirubin concentration in the drain fluid, with or without clinical symptoms, can be promptly treated with PTBD without necessitating reconstruction of the BDA.

We conducted a comprehensive literature review on bile leakage measurement after liver surgery, finding no definitive guidelines for initiating drainage therapy. Wellner and Keck suggested that a high-volume bile leak (>400 ml/24 hours) within three days post-operation indicates the need for relaparotomy [36]. Viganò et al. identified persistent bile drainage as a predictor of conservative management failure, with drainage output >100 ml on day 10 representing the only independent predictor in multivariate analysis [37]. Bhattacharjee noted that bile leakage >300 ml/24 hours is a sign of a major leak after cholecystectomy [38], and Kissen and Grundy reported relaparotomy for a patient with >300 ml/24 hours bile discharge post-cholecystectomy [39]. Ahmad et al. defined persistent drainage of 200 ml/day as indicative of a major leak [2]. Lowy et al. recommended drain removal on day three if output was <200 ml/24 hours with normal amylase [40]. Yeh et al. diagnosed leaks when bile discharge had an amylase content three times higher than serum levels [41]. Koch et al. and the International Study Group of Liver Surgery (ISGLS, see Table 4) defined bile leakage as a bilirubin concentration in drain fluid three times that of serum on or after post-operative day three [5,8].

**Table 4:** Definition and grading of bile leakage according to the International Study Group of Liver Surgery (ISGLS). [8]

Grade	Definition
A	Bile leakage requiring no or little change in patients’ clinical management
B	Bile leakage requiring a change in patients clinical management (eg, additional diagnostic or interventional procedures) but manageable without relaparotomy, or a Grade A bile leakage lasting for >1 week
C	Bile leakage requiring relaparotomy

Given the varying definitions and treatment approaches, uncertainty exists regarding the definition of a bile leak and the appropriate treatment threshold. At our pancreatic center, a consensus among surgeons, radiologists, and gastroenterologists supports therapeutic intervention for bile leaks exceeding >300 ml/24 hours within the first three days post-operation, irrespective of ISGLS grades.

Regarding PTBD placement, there is debate on the optimal timeline. While conservative management (antibiotics, surgical drains, and observation) is effective for small bile leaks, early PTBD treatment within three to seven days is recommended by Pedicini et al. and May and Hunold for persistent or worsening leaks [42,43]. Yamashita et al. intervention for leaks persisting beyond 10 days, particularly from the hilar bile duct [44], while Tanaka et al. advocated for interventional therapy after 14 days of persistent leakage [45]. In our study, all patients underwent drainage within 12.1 ± 7.3 days of bile leak detection, resulting in satisfactory outcomes. Similar trends have been observed in studies on chronic disease outcomes, reinforcing the importance of early intervention in reducing mortality risks [46].

Eight patients required additional ultrasound- or CT-guided drainage for bilioma management. Studies report technical success rates for CT-guided drainage up to 93.4%, with clinical success rates varying based on inflammatory markers [47-49]. In our cohort, additional percutaneous CT- or ultrasound-guided drainages were more commonly used in the primary PTBD group. While these drains were placed to manage perihepatic fluid collections, no conclusions can be drawn about their independent therapeutic impact, as their use was not evaluated separately.

In our study, clinical success was notably higher and mortality lower in the primary PTBD group compared to the secondary PTBD group. However, these findings should be interpreted with caution, as patients in the primary PTBD group may have been clinically less severe than those in the secondary PTBD group who had already undergone surgical revision prior to PTBD, in addition to the limitations of the retrospective design and small sample size.

The markedly higher mortality rate in the secondary PTBD group (85.7%) may reflect selection bias, as patients with more advanced clinical deterioration or complex surgical histories were more likely to be referred for early surgical intervention. Other contributing factors may include delays in initiating PTBD, underlying comorbidities, and procedural complexity. However, the limited cohort size and observational nature of the study prevent us from definitively isolating these variables.

Despite these limitations, the favorable outcomes observed in the primary PTBD group require the need to further investigate this approach as a potential first-line treatment for bile duct insufficiency following hepatobiliary surgery.

Future directions

To validate the findings of this study, future research should focus on prospective, multicenter trials comparing PTBD with surgical revision and endoscopic drainage approaches in patients with postoperative bile leaks.

These studies should aim to standardize PTBD timing, define escalation protocols, measure quality-of-life and long-term outcomes, and assess stent dependency, complication profiles, and cost-effectiveness.

Study limitations

Several limitations must be acknowledged. First, the small sample size limits the statistical power of the study and restricts the generalizability of the findings, particularly in assessing mortality differences between the primary and secondary PTBD groups. While our results suggest a significantly higher mortality risk in the secondary PTBD group, these findings require validation in larger, prospective studies. to establish definitive conclusions.

Second, although early PTBD timing may influence clinical outcomes, a subgroup analysis based on timing was not feasible due to the limited cohort size. Future studies should explore this variable in a systematic manner.

Third, a formal sample size calculation was not performed, as this was a retrospective observational study including all eligible patients treated within the defined study period.

Lastly, the heterogeneity of the patient population, with varying underlying diseases and surgical indications, may have influenced the outcomes. Standardized treatment protocols and long-term follow-up data are needed to better evaluate the durability and safety of PTBD in different clinical scenarios.

## Conclusions

This study highlights the potential role of PTBD as a minimally invasive treatment option for selected patients with postoperative bile leaks. While the results suggest favorable outcomes with PTBD, particularly in terms of clinical success and mortality, these findings are exploratory and limited by the retrospective design and small sample size. Larger, prospective studies are essential to confirm these results and establish evidence-based guidelines for the management of postoperative biliary leaks.
